# Structural and Functional Amygdala Abnormalities in Hemifacial Spasm

**DOI:** 10.3389/fneur.2019.00393

**Published:** 2019-04-30

**Authors:** Hui Xu, Chenguang Guo, Haining Li, Lin Gao, Ming Zhang, Yuan Wang

**Affiliations:** ^1^Department of Medical Imaging, The First Affiliated Hospital of Xi'an Jiaotong University, Xi'an, China; ^2^Key Laboratory of Biomedical Information Engineering of Education Ministry, Institute of Biomedical Engineering, Xi'an Jiaotong University, Xi'an, China; ^3^Faculty of Dentistry, University of Toronto, Toronto, ON, Canada

**Keywords:** hemifacial spasm, subcortical volumetric analysis, functional connectivity, facial spasm score, affective disorders

## Abstract

**Background and Purpose:** Hemifacial spasm (HFS) is a rare neuromuscular disorder characterized by unilateral, involuntary, and paroxysmal contractions of orofacial muscles. To elucidate the central neural mechanisms of HFS, we investigated brain gray matter and resting-state functional connectivity (rsFC) in HFS patients.

**Methods:** Thirty patients with HFS and 30 age- and sex-matched healthy participants consented to the study. T1-weighted structural magnetic resonance imaging (MRI) and resting-state BOLD images were collected in all participants. Cortical gray matter thickness was assessed, and subcortical volumetric analysis was performed. Seed-based rsFC analysis was performed on structurally abnormal regions in HFS patients. *Post hoc* correlations with HFS severity and measures of mood (i.e., depression and anxiety) were performed to characterize rsFC alterations.

**Results:** There were no significant differences in cortical thickness in HFS patients compared to healthy controls. Patients with HFS presented smaller right amygdala volume in contrast to healthy controls (*q* < 0.05, false-discovery rate corrected). We found that the right amygdala had increased rsFC with bilateral medial prefrontal cortex (mPFC), bilateral orbital frontal cortex (OFC), and left posterior insula (L postIns; voxel-wise *p* < 0.05, family-wise error corrected). Moreover, the connections of amygdala–postIns and amygdala–mPFC were positively related to HFS severity and anxiety, respectively.

**Conclusions:** This is the first study to show structural and functional brain abnormalities in HFS. The volumetric and rsFC amygdala abnormalities were potentially driven by HFS, providing novel insights into HFS pathophysiology.

## Introduction

Hemifacial spasm (HFS) is a neuromuscular movement disorder characterized by unilateral, involuntary, and paroxysmal contractions of the muscles innervated by the facial nerve (1, 2). The spasm usually originates from the orbicularis oculi muscle of the eyelid, and as the disease progresses, spasms spread to the orbicularis oris and buccinator muscles (1, 3). Even though HFS is not a life-threatening condition, it inevitably causes various degrees of visual and verbal disabilities, which can be distressing and lead to social phobia (4).

It is widely considered that HFS is caused by vascular contact to the facial nerve in the cerebellopontine angle cistern (5, 6); however, to date, only two studies have evaluated gray matter abnormalities in HFS patients. The first study by Bao et al. (7) found that patients with HFS showed reduced gray matter volume (GMV) in the thalamus, putamen, pallidum, amygdala, and parahippocampal gyrus compared to healthy volunteers. The second study, however, found that HFS patients had decreased GMV in the right inferior parietal lobule and increased GMV in the cerebellar lobule compared to controls (8). It should be noted that both studies employed voxel-based morphometry (VBM) to investigate GMV abnormalities (9). Surface-based analyses (SBAs) can detect thickness differences in the cortical sheets between patients and healthy controls. This method accounts for interindividual anatomical variability of the cortical surface through gyral and sulcal geometry, which can directly measure cortical thickness and areas with subvoxel precision (10). This increases the sensitivity to gray matter abnormalities. To the best of our knowledge, no study has applied SBA to determine whether there are cortical thickness abnormalities in HFS patients. However, SBA is limited to cortical regions, and thus, subcortical structures must be evaluated through other volumetric means.

Resting-state functional connectivity (rsFC) is a functional magnetic resonance imaging (fMRI) method used to probe temporal correlations in spontaneous, low-frequency fluctuations across functionally related but structurally distinct brain regions without designated tasks (11). Furthermore, this technique is also suitable for revealing the functional reorganization in intrinsic brain networks in various pathological states (12, 13). To date, no studies have investigated rsFC in HFS. The only study to detect functional abnormalities in HFS investigated signal coherence [or regional homogeneity (ReHo)], a measure of time series similarity in a voxel and its neighbors. The authors found that patients with HFS showed decreased ReHo values in the middle frontal gyrus (MFG) and middle cingulate cortex (MCC), and increased ReHo in the precentral gyrus and brainstem (14). Nevertheless, they were unable to investigate the rsFC alterations in the brain associated with structural abnormalities in the patient group. Besides, it remains unknown whether alterations of gray matter or rsFC are related to disease characteristics and mood disorders, such as anxiety and depression in HFS. It is possible that the social phobia experienced by patients may be linked to brain abnormalities.

Therefore, the objectives of this study are to (1) identify cortical thickness and subcortical volume changes in HFS patients and (2) determine whether these structural abnormalities are related to rsFC abnormalities. Next, we will test whether these abnormalities are associated with the severity of disease and degree of mood disorders.

## Methods

### Participants and Neuropsychological Assessment

Thirty primary HFS patients were recruited from the department of neurology at the First Affiliated Hospital of Xi'an Jiaotong University. Inclusion criteria were as follows: disease duration >6 months and typical hemifacial muscle spasms with involuntary and intermittent onset, as independently diagnosed by two experienced physicians. Exclusion criteria included secondary HFS caused by tumors and cysts, organic brain disorders, significant premorbid psychiatric or neurological history, no history of microvascular decompression surgery or botulinum neurotoxin injection, and contraindication to MRI scans (e.g., claustrophobia). Thirty age- and sex-matched healthy volunteers also enrolled in this study. Participants had no history of psychiatric or neurological illness, and no history of alcohol or drug abuse. Written informed consent was obtained from all subjects prior to participation in accordance with the Declaration of Helsinki.

All subjects underwent a structured clinical interview and completed a brief psychological assessment, including the Hamilton Depression Scale (HAM-D) and Hamilton Anxiety Scale (HAM-A). In addition, patients with HFS were also assessed by the Cohen evaluation scale to quantify severity of facial muscle spasms [0–4 scale: 0 = none; 1 = increased blinking caused by external stimuli; 2 = mild, noticeable fluttering, not incapacitating; 3 = moderate, very noticeable spasm, mildly incapacitating; 4 = severely incapacitating (unable to drive, read, etc.)] (15).

### Structural and Functional Magnetic Resonance Imaging Data Acquisition

Neuroimaging data from patients with HFS and healthy controls were acquired using the GE Signa HDxt 3.0-T MRI system with an eight-channel head coil. Three-dimensional anatomical images were acquired using a magnetization-prepared rapid acquisition gradient echo (MPRAGE) sequence [time of repetition (TR) = 10.7 ms, time of echo (TE) = 4.9 ms, flip angle (FA) = 15°, in-plane resolution = 1 × 1 × 1 mm, matrix size = 256 × 256, field of view (FOV) = 256 × 256 mm, scan duration = 4 min and 51 s]. Next, a resting-state fMRI scan was collected for each subject using gradient echo–echo planar imaging (GRE-EPI; 150 volumes per slice, TR = 2,000 ms, TE = 35 ms, FA = 90°, in-plane resolution = 3.75 × 3.75 × 4 mm, matrix size = 64 × 64, FOV = 240 × 240 mm). Participants were asked to keep their eyes closed and to remain awake during resting-state fMRI. Finally, diffusion-tensor imaging was also collected after the T1-weighted and resting-state fMRI, but was not discussed in this study.

### Measurements of Cortical Thickness and Subcortical Volumes

Each T1-weighted MRI was processed using FreeSurfer (version 5.3.0, http://surfer.nmr.mgh.harvard.edu) with its standard processing pipeline to generate cortical surface models and measure cortical thickness and subcortical volumes. Briefly, for each T1-weighted volume, gray and white matter tissues were segmented, followed by a three-dimensional reconstruction of the gray matter surface and the cortical mantle. The cortical sheet is now represented by vertices, rather than voxels, as it is represented by a surface. Then, cortical thickness at every vertex was determined by computing the distance between the boundary of white matter and the pial boundaries of the gray matter surface. Surface maps were generated following registration of all individuals' cortical reconstructions to a common average template. Finally, surface maps were smoothed with a 10-mm full width at half maximum (FWHM) Gaussian kernel.

Whole-brain vertex-wise analysis of cortical thickness was performed using the Qdec module implemented in FreeSurfer, with a general linear model (GLM) examining group differences. Maps showing significant group differences between patients and healthy controls were generated by thresholding the images of *t* statistics with false-discovery rate (FDR) correction of *p* < 0.05 at cluster level followed by a cluster-forming threshold of *p* < 0.001, marking the cortical regions with significant changes. In addition, a volumetric analysis of subcortical structures was performed based on the FreeSurfer subcortical segmentation pipeline. These volumes were compared using *t* tests and further corrected for multiple comparisons using FDR control with a *q* < 0.05.

### Resting-State Functional Magnetic Resonance Imaging Connectivity

The resting-state fMRI data were analyzed using FSL (Version 5.0) and included removal of the first five volumes, slice timing and head motion correction, realignment, spatial normalization (to MNI space), spatial smoothing using an 8-mm isotropic Gaussian kernel, temporal band-pass filtering (0.01–0.1 Hz), and elimination of nuisance signals including head motion parameters from MCFLIRT (part of FSL), white matter signal, and cerebrospinal fluid signal by exacting their mean time series.

To examine rsFC changes related to the morphological abnormalities in HFS patients, regions with significant between-group differences of cortical thickness or subcortical volumes were extracted as seed regions. The subcortical seed region was defined by getting the 95% maximum intensity value of this region of the Harvard–Oxford Subcortical Structural Atlas in the standard MNI template space. Then, correlation coefficients between the mean time series of each seed and time series of every voxel throughout the rest of the brain were calculated as rsFC map, which was further converted to *z* values using Fisher's *z* transformation to improve normality. A permutation-based two-sample *t* test was run to generate group-level-difference maps of rsFC for each seed region and then corrected for multiple comparisons with a family-wise error (FWE) rate of *p* < 0.05.

### Quality Control of Structural and Functional Magnetic Resonance Imaging

During the structural and functional MRI analysis, we inspected any artifact that could affect processing, including segmentation, normalization, etc. In addition, 7 subjects (5 patients and 2 controls) with head motion of any volume more than 1.5 mm or 1.5° were excluded in further MRI data analysis, leaving a total of 53 participants (25 patients and 28 controls, details seen in Table 1) in this study.

**Table 1:** Summary of demographic characteristics and psychiatric tests between patients with HFS and healthy controls.

**Characteristic**	**HFS (*n* = 25)**	**HC (*n* = 28)**	***p*-value**
Age (years)	48.32 ± 11.59	48.96 ± 12.27	>0.05
Sex			>0.05
Male	11	12	>0.05
Female	14	16	
Disease duration (years)	3.61 ± 3.55	N.A.	
Cohen scores	2.93 ± 0.77	N.A.	
Psychiatric tests			
HAM-A scores	5.21 ± 2.73	0.33 ± 0.91	0.000^*^
HAM-D scores	5.04 ± 2.85	0.28 ± 0.90	0.000^*^

*HFS, hemifacial spasm; HC, healthy controls; Cohen scores, spasm severity rating via the Cohen evaluation scale; HAM-A, Hamilton Anxiety; HAM-D, Hamilton Depression; N.A., not assigned. Values given as mean ± standard deviation*.

* *Significant difference between groups*.

### Association of Functional Connectivity to Clinical Indices in Patients With Hemifacial Spasm

Spearman correlation coefficients were calculated to evaluate the relationship between clinical variables (Cohen evaluation scale, HAMD score, and HAMA score) and functional connectivity values from group-level-difference clusters of rsFC analysis using SPSS software version 18.0. A *p*-value of <0.05 was considered statistically significant after correction for multiple comparisons with Bonferroni test.

## Results

### Demographics and Neuropsychological Assessment

Patients with HFS and healthy controls were matched well for age (48.32 ± 11.59 years old for patients and 48.96 ± 12.27 years old for controls, *t*_51_ = −0.195, *p* = 0.846) and sex (56.0% female patients vs. 57.1% female controls, $\chi_{1}^{2}$ = 0.007, *p* = 0.933). In addition, patients with HFS reported significant levels of anxiety (*t*_51_ = 8.924, *p* < 0.001) and felt more depressed (*t*_51_ = 8.110, *p* < 0.001) than healthy controls, which were measured by HAM-A and HAM-D, respectively. Demographic and clinical data are all presented in Table 1.

### Abnormal Cortical Thickness and Subcortical Volumes in Patients With Hemifacial Spasm

**Figure 1:**
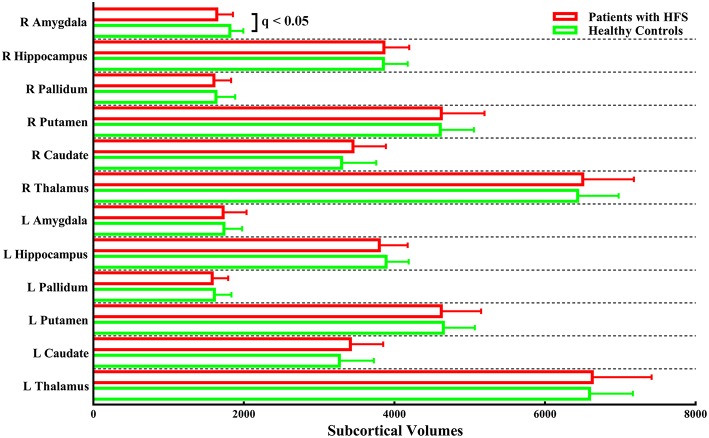
Subcortical volumes in patients with HFS compared with healthy controls. Patients merely showed significantly reduced subcortical volume in the right amygdala compared to the control group [*q* < 0.05, false-discovery rate (FDR) corrected for multiple comparisons]. L, left; R, right.

The unbiased whole-brain vertex-wise comparison showed no significant differences that survived multiple comparisons (FDR correction of *p* < 0.05 at a cluster level followed by a cluster-forming threshold of *p* < 0.001) in patients with HFS compared with healthy controls. Furthermore, the volumetric analysis of subcortical structures showed significantly reduced subcortical volume merely in the right amygdala in patients with HFS compared to healthy controls (*q* < 0.05, FDR corrected; Figure 1).

### Abnormal Right Amygdala-Based Functional Connectivity Following Patients With Hemifacial Spasm

**Figure 2:**
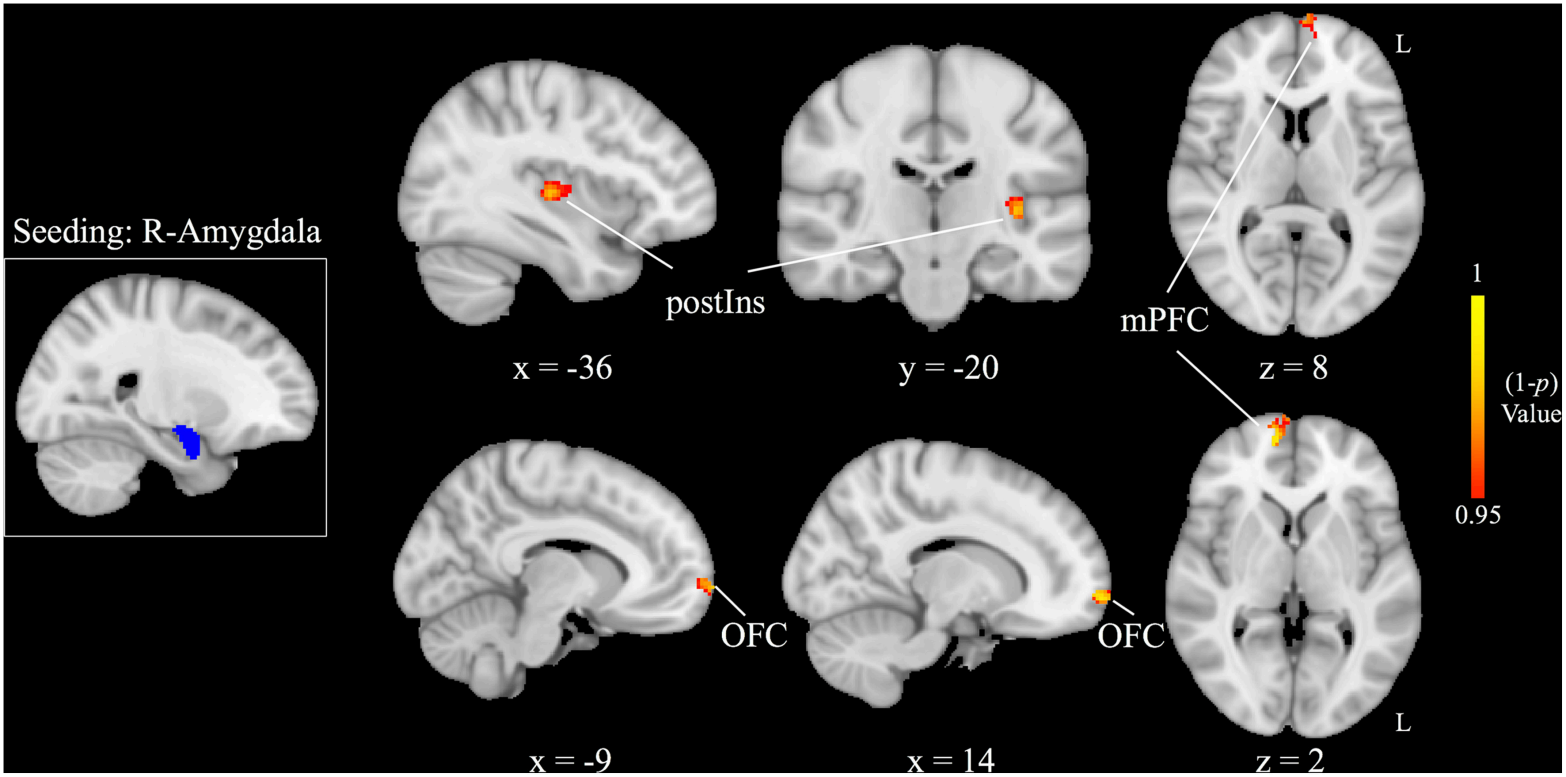
Increased right amygdala-based functional connectivity following patients with hemifacial spasm (HFS) in contrast to healthy controls [ *p* < 0.05, family-wise error (FWE) corrected]. postIns, posterior insula; mPFC, medial prefrontal cortex; OFC, orbital frontal cortex; L, left; R, right.

Increased right amygdala-anchored rsFC to the bilateral medial prefrontal cortex (mPFC), bilateral orbital frontal cortex (OFC), and left posterior insula (L postIns) was observed in patients with HFS compared with healthy controls (*p* < 0.05, FWE corrected; Figure 2, Table 2).

**Table 2:** Clusters demonstrating differences in functional connectivity between HFS and HC participants.

**ROI**	**Brain region**	**Hemisphere**	**Size of cluster (voxels)**	**Peak MNI coordinate**			**Peak voxel *t-*value**
				**x**	**y**	**z**	
**HFS** **>** **HC**							
Right amygdala	Medial prefrontal cortex	L	137	−8	70	12	3.625
	Medial prefrontal cortex	R	370	12	56	2	3.434
	Orbital frontal cortex	L	52	−8	70	−2	3.298
	Orbital frontal cortex	R	62	14	68	−6	3.315
	Posterior insula	L	100	−36	−20	4	4.758
**HFS** **<** **HC**							
No between-group differences							

*ROI, region of interest; MNI, Montreal Neurologic Institute; L, left; R, right*.

### Association of Functional Connectivity to Clinical Variables in Patients With Hemifacial Spasm

**Figure 3:**
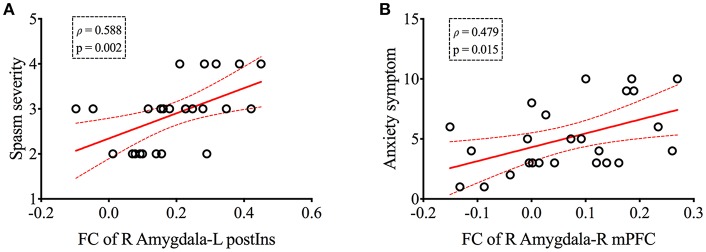
The right amygdala-based functional connectivity was positively correlated with spasm severity **(A)** and anxiety symptom **(B)** in patients with HFS, respectively. Curved dashed lines indicate 95% confidence intervals. The spasm severity was measured by Cohen evaluation scale, and Hamilton Anxiety Scale was performed to assess patients' anxiety symptom. FC, functional connectivity; postIns, posterior insula; mPFC, medial prefrontal cortex; L, left; R, right.

Correlations of FC values from group-level-difference clusters of rsFC were detected to the clinical indices of patients with HFS. We found that the mean FC value of right amygdala to L postIns positively correlated with spasm severity (ρ = 0.588, *p* = 0.002, Figure 3A). In addition, the mean FC value of the right amygdala to right mPFC also correlated with anxiety symptom (ρ = 0.479, *p* = 0.015, Figure 3B). No other significant correlation was found between FC values and other clinical parameters (i.e., disease duration and HAM-D score).

## Discussion

This study investigated structural and functional reorganization in the brain associated with HFS and how these changes are relevant to the severity of muscle contraction and concomitant affective disturbance. Patients with HFS had significantly reduced volume in the right amygdala. Furthermore, compared with healthy controls, the right amygdala displayed increased rsFC to the bilateral mPFC, bilateral OFC, and L postIns in patients with HFS. To date, there has been only one resting-state fMRI study related to HFS, which focused on evaluating synchronous brain activity of a given region to its nearest neighbors by ReHo analysis (14) without capturing the functional relationships to the distant brain areas. To our knowledge, this is the first study to show structural and functional amygdala abnormalities in HFS.

One of the major findings of this study presented as decreased volume of right amygdala in patients with HFS compared to healthy controls. The amygdala belongs to a key region with afferent and efferent neural connections that modulate complex stimuli such as pain, anxiety, fear, and reward (16). Corresponding to its functional diversity, the amygdaloid complex has been shown to consist of dozens of distinct but closely interconnected nuclei in nonhuman primates (17). In addition, cytoarchitectonic study of human postmortem brains suggested that amygdala includes three major sets of nuclei that are called laterobasal, centromedial, and superficial groups (18). For one, the centromedial nuclei of amygdala can produce hormones and induce autonomic responses according to the anatomical and physiological knowledge (19–22), which involves the process of unpleasant stimuli including anxious and depressive information (23, 24), and the spasm-anchored affective disorders in patients with HFS may partially contribute to the amygdala atrophy. For another, the morphological and functional alterations of amygdala may be linked to visual attentional deficit triggered by the spasm in patients with HFS. The lateral portion of amygdala was established to coordinate visual information, which was supported by a tract-tracing study (25) and single-cell recordings (26) in monkeys. Besides, bidirectional communication between amygdala and fusiform gyrus was further verified during facial information processing by fMRI experiment (27). Given that those enrolled in this study underwent chronic and severe facial spasm (mean Cohen score was near 3), patients with HFS were difficult to concentrate on others' face in a short period of time during social contact, which probably lead to the structural alteration in the laterobasal nuclei of amygdala. Since anatomical tracing studies revealed that most of the nuclei in the amygdaloid complex had extensive intranuclear and internuclear connections (22), our imaging data on amygdala abnormality may be attributed to the interaction of emotional and visual deficiency in patients with HFS.

Another finding in our study was altered right amygdala-driven connections to several emotion-related brain areas, such as bilateral mPFC and bilateral OFC in patients with HFS compared to healthy controls, which was in accordance with previous fMRI results (28). Previous animal and human studies suggested a distinctive amygdala–frontal circuit on emotion generation and regulation (29–31). Anatomical tracing studies have detected reciprocal connections between the amygdala and the anterior cingulate cortex (ACC), OFC, and dorsal medical prefrontal cortex (DMPFC) (32, 33). Of note, the OFC was usually segmented into medial and lateral divisions, which initially derived from differential cognitive and affective deficits of medial OFC (mOFC) vs. lateral OFC (lOFC) in primates (34), and this parcellation was confirmed by differentiated connections with tracing studies; that is, the mOFC received inputs from limbic structures such as hippocampus, amygdala, and insular cortex (35), while lOFC showed anatomic connections with several visual processing regions including fusiform gyrus and lateral occipital cortex (36). Moreover, in a meta-analysis focusing on divergent patterns of rsFC between different OFC subregions, the lOFC showed notable coactivations with the amygdala and the fusiform gyrus, both of which are known to participate in visual processing (37). Accordingly, we assume that the strengthened rsFC of amygdala to lOFC may be helpful for patients with HFS to compensate for the deficit of focusing on objects for a long period of time.

Meanwhile, our study displayed increased right amygdala rsFC to bilateral mPFC in patients with HFS. The mPFC was involved in various categories of affective disorders detected by fMRI and PET, such as schizophrenia, bipolar disorder (38), major depression (39), and social anxiety (40). Electron microscopy (41) and retrograde tracing techniques (42, 43) confirmed that the mPFC directly received extensive input from amygdala, which was important for emotion regulation. Considering that the current findings demonstrated positive correlation of amygdala–mPFC connection to the degree of anxiety in patients with HFS, we proposed that the rsFC abnormality of the two regions in the patient group was caused in part by the spasm-induced negative emotion. In addition, it is well known that mPFC is a major hub of default mode network. Because mPFC activity has linked to maintain vigilance toward the surrounding environment (44), and the laterobasal nuclei group in amygdala was established to coordinate high-level sensory input including visual information preprocessing (17), the increased connectivity between mPFC and amygdala could also reflect exaggerated vigilance to the environment so as to compensate for the deficit of visual attention in patients with HFS.

Last but not the least, the patients with HFS exhibited increased amygdala–postIns connection compared to controls. It is well established that insula has structural and functional connections with almost all of the amygdaloid nuclei. Neurophysiological and histochemical experiments had confirmed projections of anterior insula to the anterior and medial amygdaloid area and projections of posterior insula to the dorsolateral part of amygdala in the rhesus monkey (45), where the corresponding nuclei are responsible for modulation of autonomic activity and high-level sensory information, respectively (17), and it was partially supported by a functional imaging study with positron emission tomography (46). Because the posterior insula and lateral part of amygdala share a functional similarity on visual stimuli coordination, increased rsFC of the two regions is likely to make up the visual defect of HFS with frequent facial contraction, which provides a basis for positive correlation of amygdala–insular connectivity to the degree of facial spasm.

Several limitations of this study bear acknowledgment here. First, the sample size of both groups is relatively small. The reliability of the results would be improved by recruiting more subjects. Second, the cross-sectional experiment design was not useful to observe dynamic changes in structural and functional dataset to assess the characteristics of HFS over time. A longitudinal study should be performed to monitor the volume and rsFC of right amygdala along with alterations of the facial spasm and emotional disturbance.

In summary, the current study revealed atrophic right amygdala in patients with HFS together with increased rsFC of this seed to bilateral OFC, bilateral mPFC, and left postIns. Moreover, the altered amygdala–postIns and amygdala–mPFC networks were correlated with spasm severity and anxiety symptom, which provide distinct aspects of clues of HFS-related disorders.

## Ethics Statement

This study was carried out in accordance with the recommendations of the Medical Research Ethics Committee and Institutional Review Board of the first affiliated hospital of Xi'an Jiaotong University with written informed consent from all subjects. All subjects gave written informed consent in accordance with the Declaration of Helsinki. The protocol was approved by the Department of medical imaging, the first affiliated hospital of Xi'an Jiaotong University.

## Author Contributions

YW, MZ, and HX drafted the manuscript, study concept or design, and statistical analysis. HX, HL, and LG revised the manuscript for content and analysis or interpretation of data. CG and HL acquired the data. YW, MZ, and HX supervised or coordinated the experiment. All authors read and approved the final manuscript.

### Conflict of Interest Statement

The authors declare that the research was conducted in the absence of any commercial or financial relationships that could be construed as a potential conflict of interest.
